# 1-Phenyl­piperazine-1,4-diium bis­(hydrogen sulfate)

**DOI:** 10.1107/S1600536810037001

**Published:** 2010-09-25

**Authors:** Houda Marouani, Mohamed Rzaigui, Salem S. Al-Deyab

**Affiliations:** a Laboratoire de Chimie des Matériaux, Faculté des Sciences de Bizerte, 7021 Zarzouna Bizerte, Tunisia; b Petrochemical Research Chair, College of Science, King Saud University, Riadh, Saudi Arabia

## Abstract

In the title compound, C_10_H_16_N_2_^2+^·2HSO_4_^−^, the S atoms adopt slightly distorted tetra­hedral geometry and the diprotonated piperazine ring adopts a chair conformation. In the crystal, the 1-phenyl­piperazine-1,4-diium cations are anchored between chains formed by the sulfate entities *via* inter­molecular bifurcated N—H⋯(O,O) and weak C—H⋯O hydrogen bonds. These hydrogen bonds contribute to the cohesion and stability of the network of the crystal structure.

## Related literature

For pharmacological properties of phenyl­piperazine, see: Cohen *et al.* (1982 ▶); Conrado *et al.* (2008 ▶); Neves *et al.* (2003 ▶). For related structures, see: Ben Gharbia *et al.* (2005 ▶). For a discussion on hydrogen bonding, see: Brown (1976 ▶); Blessing (1986 ▶). For structural discussion, see: Arbuckle *et al.* (2009 ▶). For puckering parameters, see: Cremer & Pople (1975 ▶).
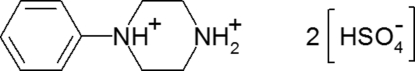


## Experimental

### 

#### Crystal data

C_10_H_16_N_2_^2+^·2HSO_4_^−^
*M* *_r_* = 358.38
Monoclinic, 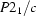

*a* = 17.535 (6) Å
*b* = 10.996 (2) Å
*c* = 7.631 (2) Å
β = 99.86 (2)°
*V* = 1449.7 (7) Å^3^
*Z* = 4
Ag *K*α radiation
λ = 0.56083 Å
μ = 0.22 mm^−1^
*T* = 293 K
0.5 × 0.4 × 0.1 mm

#### Data collection

Enraf–Nonius CAD-4 diffractometer
8212 measured reflections
7100 independent reflections
4535 reflections with *I* > 2σ(*I*)
*R*_int_ = 0.022
2 standard reflections every 120 min  intensity decay: 1%

#### Refinement

*R*[*F* ^2^ > 2σ(*F* ^2^)] = 0.046
*wR*(*F* ^2^) = 0.127
*S* = 1.03
7100 reflections
201 parameters
H-atom parameters constrained
Δρ_max_ = 0.43 e Å^−3^
Δρ_min_ = −0.46 e Å^−3^

### 

Data collection: *CAD-4 EXPRESS* (Enraf–Nonius, 1994 ▶); cell refinement: *CAD-4 EXPRESS*; data reduction: *XCAD4* (Harms & Wocadlo, 1995 ▶); program(s) used to solve structure: *SHELXS86* (Sheldrick, 2008 ▶); program(s) used to refine structure: *SHELXL97* (Sheldrick, 2008 ▶); molecular graphics: *ORTEP-32* for Windows (Farrugia, 1998 ▶); software used to prepare material for publication: *WinGX* (Farrugia, 1999 ▶).

## Supplementary Material

Crystal structure: contains datablocks I, global. DOI: 10.1107/S1600536810037001/pv2328sup1.cif

Structure factors: contains datablocks I. DOI: 10.1107/S1600536810037001/pv2328Isup2.hkl

Additional supplementary materials:  crystallographic information; 3D view; checkCIF report

## Figures and Tables

**Table 1:** Hydrogen-bond geometry (Å, °)

*D*—H⋯*A*	*D*—H	H⋯*A*	*D*⋯*A*	*D*—H⋯*A*
O1—H1⋯O3^i^	0.82	1.80	2.6140 (17)	172
O5—H5⋯O7^ii^	0.82	1.80	2.6066 (18)	169
N1—H1*A*⋯O2^iii^	0.90	2.23	2.8636 (19)	128
N1—H1*A*⋯O3^iv^	0.90	2.30	3.0279 (19)	138
N1—H1*B*⋯O3	0.90	2.14	2.9251 (18)	145
N1—H1*B*⋯O2^i^	0.90	2.35	2.9892 (18)	128
N2—H2⋯O7	0.91	2.02	2.8216 (16)	146
N2—H2⋯O6^ii^	0.91	2.32	2.9037 (17)	122

Symmetry codes: (i) 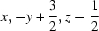; (ii) 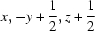; (iii) 
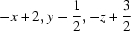
; (iv) 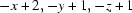.

**Fig. 1.**
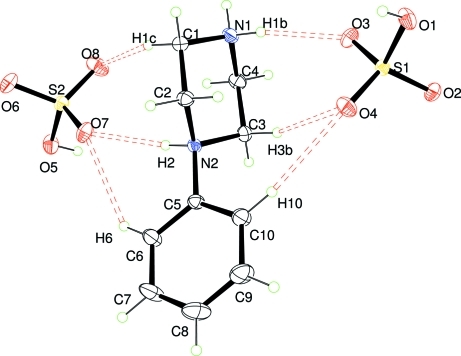
An ORTEP view of (I) with the atom-labelling scheme. Displacement ellipsoids are drawn at the 30% probability level. H atoms are represented as small spheres of arbitrary radii.

**Fig. 2.**
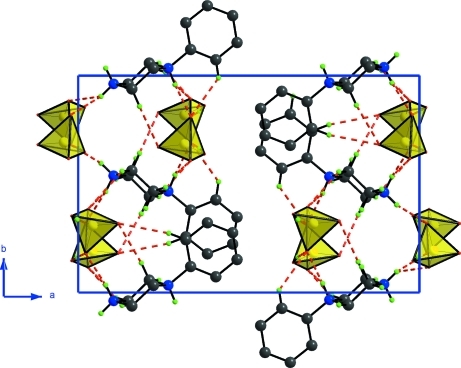
Projection of (I) along the c axis. H atoms non committed in H-bonds are omitted for clarity.

## supplementary crystallographic information

### Comment

The phenylpiperazine and its derivatives have been intensively investigated recently owing to their interesting pharmacological, cardiovascular and autonomic properties (Conrado *et al.*, 2008; Cohen *et al.*, 1982; Neves *et al.*, 2003). We report here the preparation and the crystal structure of the title compound, (I).

The asymmetric unit of the title compound (Fig.1) consists of two HSO_4_^-^ anions and a 1-phenylpiperazine-1,4-diium cation. The interatomic bond lengths and angles of the cation show no significant deviation from those reported in other 1-phenylpiperazinium salts such as [C_10_H_16_N_2_]_2_ZnCl_4_ (Ben Gharbia, *et al.*, 2005). In the title compound, the distances S—O are significantly longer than the S═O distances as reported in the hydrogen sulfate ion previously (Arbuckle, *et al.*, 2009). The aromatic ring is essentially planar while the diprotonated piperazine ring adopts a chair conformation, with puckering parameters (Cremer & Pople, 1975): *Q* = 0.5913 (14) Å, θ = 178.61 (15)° and φ = 76 (5)°.

The atomic arrangement is characterized by infinite chains built by HSO_4_^-^ anions. The inorganic chains, extending along the *c* direction, are located around planes perpendicular to the *a* axis at *x* = 0 (for HS1O_4_^-^) and *x* = 1/4, *x* = 3/4 (for HS2O_4_^-^). The hydrogen sulfate groups of the same type are interconnected *via* strong O—H···O hydrogen bonds (Table 1)[d (O···O) < 2.73 Å] (Brown, 1976; Blessing, 1986). Chains formed by HS1O_4_ are linked by N1 nitrogen atom of the cation to form layers parallel to the *bc* plane at *x* = 0. Two chains of different type are bound between them by the cations through their two nitrogen atoms by means of the N—H···O hydrogen bonds (Fig. 2).

The cations are linked onto the anionic chains, by forming H-bonds with the oxygen atoms with N—H···O distances in the range 2.8216 (16)–3.0279 (19) Å and C—H···O distances in the range 2.949 (2)–3.520 (2) Å. It should be noticed that all the amino hydrogen atoms are involved in bifurcated N—H···(O, *O*) hydrogen bonding. These hydrogen bonds contribute to the cohesion and stability of the network of the studied crystal structure.

### Experimental

Single crystals of the title compound were prepared at room temperature from a mixture of an aqueous solution of sulfuric acid (2 mmol), 1-phenylpiperazine (1 mmol), ethanol (10 ml) and water (10 ml). The solution was stirred for 1 h then evaporated slowly at room temperature for several days until the formation of good quality of prismatic single crystals.

### Refinement

All H atoms were fixed geometrically and treated as riding with C—H = 0.93 Å (aromatic) or 0.97 Å (methylene), N—H = 0.90 Å or 0.91 Å and O—H = 0.82 Å with *U*_iso_(H) = 1.2*U*_eq_(C or N) or 1.5*U*_eq_(O).

### Crystal data

**Table:**

C_10_H_16_N_2_^2+^·2HSO_4_^−^	*F*(000) = 752
*M**_r_* = 358.38	*D*_x_ = 1.642 Mg m^−^^3^
Monoclinic, *P*2_1_/*c*	Ag *K*α radiation, λ = 0.56083 Å
Hall symbol: -P 2ybc	Cell parameters from 25 reflections
*a* = 17.535 (6) Å	θ = 9–11°
*b* = 10.996 (2) Å	µ = 0.22 mm^−^^1^
*c* = 7.631 (2) Å	*T* = 293 K
β = 99.86 (2)°	Prism, colorless
*V* = 1449.7 (7) Å^3^	0.5 × 0.4 × 0.1 mm
*Z* = 4	

### Data collection

**Table:**

Enraf–Nonius CAD-4 diffractometer	*R*_int_ = 0.022
Radiation source: fine-focus sealed tube	θ_max_ = 28.0°, θ_min_ = 2.4°
graphite	*h* = −3→29
non–profiled ω scans	*k* = −18→0
8212 measured reflections	*l* = −12→12
7100 independent reflections	2 standard reflections every 120 min
4535 reflections with *I* > 2σ(*I*)	intensity decay: 1%

### Refinement

**Table:**

Refinement on *F*^2^	Primary atom site location: structure-invariant direct methods
Least-squares matrix: full	Secondary atom site location: difference Fourier map
*R*[*F*^2^ > 2σ(*F*^2^)] = 0.046	Hydrogen site location: inferred from neighbouring sites
*wR*(*F*^2^) = 0.127	H-atom parameters constrained
*S* = 1.03	*w* = 1/[σ^2^(*F*_o_^2^) + (0.0603*P*)^2^ + 0.1738*P*] where *P* = (*F*_o_^2^ + 2*F*_c_^2^)/3
7100 reflections	(Δ/σ)_max_ = 0.001
201 parameters	Δρ_max_ = 0.43 e Å^−^^3^
0 restraints	Δρ_min_ = −0.46 e Å^−^^3^

### Special details

**Table:**

Geometry. All e.s.d.'s (except the e.s.d. in the dihedral angle between two l.s. planes) are estimated using the full covariance matrix. The cell e.s.d.'s are taken into account individually in the estimation of e.s.d.'s in distances, angles and torsion angles; correlations between e.s.d.'s in cell parameters are only used when they are defined by crystal symmetry. An approximate (isotropic) treatment of cell e.s.d.'s is used for estimating e.s.d.'s involving l.s. planes.
Refinement. Refinement of *F*^2^ against ALL reflections. The weighted *R*-factor *wR* and goodness of fit *S* are based on *F*^2^, conventional *R*-factors *R* are based on *F*, with *F* set to zero for negative *F*^2^. The threshold expression of *F*^2^ > σ(*F*^2^) is used only for calculating *R*-factors(gt) *etc*. and is not relevant to the choice of reflections for refinement. *R*-factors based on *F*^2^ are statistically about twice as large as those based on *F*, and *R*- factors based on ALL data will be even larger.

### Fractional atomic coordinates and isotropic or equivalent isotropic displacement parameters (Å^2^)

**Table:**

	*x*	*y*	*z*	*U*_iso_*/*U*_eq_	
S1	0.958198 (19)	0.80824 (3)	0.59663 (4)	0.02129 (8)	
S2	0.68841 (2)	0.18262 (3)	0.36929 (5)	0.02431 (8)	
O7	0.66990 (8)	0.31003 (10)	0.32493 (15)	0.0332 (2)	
O3	0.97861 (7)	0.68051 (10)	0.64169 (14)	0.0306 (2)	
N2	0.73629 (7)	0.53363 (10)	0.45259 (15)	0.0215 (2)	
H2	0.7163	0.4579	0.4607	0.026*	
O2	0.97180 (7)	0.88315 (10)	0.75428 (14)	0.0295 (2)	
N1	0.89625 (7)	0.46159 (12)	0.49684 (18)	0.0273 (2)	
H1A	0.9333	0.4041	0.5136	0.033*	
H1B	0.9194	0.5342	0.4913	0.033*	
O1	1.01876 (7)	0.85308 (12)	0.48419 (14)	0.0355 (3)	
H1	1.0036	0.8368	0.3791	0.053*	
O4	0.88248 (7)	0.81856 (12)	0.49109 (16)	0.0366 (3)	
O5	0.63020 (7)	0.13868 (12)	0.48817 (15)	0.0356 (3)	
H5	0.6438	0.1639	0.5898	0.053*	
O6	0.67139 (8)	0.10676 (11)	0.21389 (16)	0.0371 (3)	
C3	0.79171 (8)	0.55729 (14)	0.62253 (19)	0.0258 (3)	
H3A	0.7639	0.5567	0.7220	0.031*	
H3B	0.8152	0.6368	0.6173	0.031*	
C2	0.77975 (9)	0.53263 (14)	0.29912 (19)	0.0266 (3)	
H2A	0.8023	0.6122	0.2876	0.032*	
H2B	0.7442	0.5155	0.1900	0.032*	
C5	0.67057 (8)	0.61993 (13)	0.42348 (19)	0.0244 (3)	
C1	0.84293 (8)	0.43819 (14)	0.3260 (2)	0.0284 (3)	
H1C	0.8203	0.3578	0.3284	0.034*	
H1D	0.8716	0.4412	0.2281	0.034*	
C4	0.85375 (9)	0.46091 (15)	0.6493 (2)	0.0293 (3)	
H4A	0.8895	0.4765	0.7588	0.035*	
H4B	0.8304	0.3817	0.6588	0.035*	
O8	0.76519 (7)	0.17042 (14)	0.47005 (18)	0.0465 (3)	
C10	0.68447 (10)	0.74340 (14)	0.4204 (3)	0.0346 (3)	
H10	0.7348	0.7735	0.4384	0.042*	
C6	0.59670 (9)	0.57280 (16)	0.3966 (2)	0.0347 (3)	
H6	0.5887	0.4892	0.3987	0.042*	
C9	0.62142 (12)	0.82154 (17)	0.3896 (3)	0.0468 (5)	
H9	0.6293	0.9051	0.3871	0.056*	
C8	0.54742 (13)	0.7761 (2)	0.3629 (3)	0.0532 (5)	
H8	0.5055	0.8291	0.3422	0.064*	
C7	0.53462 (10)	0.6521 (2)	0.3664 (3)	0.0489 (5)	
H7	0.4843	0.6221	0.3485	0.059*	

### Atomic displacement parameters (Å^2^)

**Table:**

	*U*^11^	*U*^22^	*U*^33^	*U*^12^	*U*^13^	*U*^23^
S1	0.02138 (14)	0.02425 (15)	0.01794 (14)	−0.00207 (12)	0.00249 (10)	−0.00047 (11)
S2	0.02718 (16)	0.02462 (15)	0.02126 (15)	−0.00051 (13)	0.00451 (12)	0.00042 (12)
O7	0.0483 (7)	0.0228 (5)	0.0284 (5)	−0.0016 (5)	0.0067 (5)	−0.0006 (4)
O3	0.0446 (6)	0.0249 (5)	0.0219 (5)	0.0038 (5)	0.0047 (4)	0.0000 (4)
N2	0.0196 (5)	0.0189 (5)	0.0255 (5)	−0.0014 (4)	0.0029 (4)	0.0000 (4)
O2	0.0372 (6)	0.0288 (5)	0.0225 (5)	−0.0006 (4)	0.0055 (4)	−0.0059 (4)
N1	0.0198 (5)	0.0266 (6)	0.0352 (6)	−0.0001 (4)	0.0037 (5)	0.0005 (5)
O1	0.0341 (6)	0.0512 (7)	0.0225 (5)	−0.0180 (5)	0.0087 (4)	−0.0030 (5)
O4	0.0250 (5)	0.0474 (7)	0.0343 (6)	−0.0012 (5)	−0.0036 (4)	0.0024 (5)
O5	0.0398 (6)	0.0412 (7)	0.0275 (5)	−0.0149 (5)	0.0103 (5)	−0.0031 (5)
O6	0.0545 (8)	0.0298 (5)	0.0286 (5)	0.0002 (5)	0.0116 (5)	−0.0078 (4)
C3	0.0247 (6)	0.0278 (6)	0.0241 (6)	−0.0011 (5)	0.0015 (5)	−0.0018 (5)
C2	0.0252 (6)	0.0316 (7)	0.0234 (6)	0.0017 (5)	0.0052 (5)	0.0003 (5)
C5	0.0221 (6)	0.0232 (6)	0.0279 (6)	0.0029 (5)	0.0040 (5)	0.0030 (5)
C1	0.0243 (6)	0.0297 (7)	0.0320 (7)	−0.0002 (6)	0.0068 (5)	−0.0057 (6)
C4	0.0251 (6)	0.0347 (8)	0.0273 (7)	0.0015 (6)	0.0019 (5)	0.0049 (6)
O8	0.0297 (6)	0.0652 (9)	0.0419 (7)	0.0035 (6)	−0.0015 (5)	0.0122 (7)
C10	0.0301 (8)	0.0244 (7)	0.0494 (10)	0.0011 (6)	0.0071 (7)	0.0036 (6)
C6	0.0234 (7)	0.0330 (8)	0.0467 (9)	−0.0006 (6)	0.0029 (6)	0.0045 (7)
C9	0.0468 (11)	0.0280 (8)	0.0661 (13)	0.0109 (8)	0.0110 (10)	0.0070 (8)
C8	0.0408 (10)	0.0490 (11)	0.0707 (15)	0.0220 (9)	0.0123 (10)	0.0172 (11)
C7	0.0211 (7)	0.0548 (12)	0.0698 (14)	0.0061 (8)	0.0050 (8)	0.0140 (11)

### Geometric parameters (Å, °)

**Table:**

S1—O4	1.4347 (13)	C3—H3B	0.9700
S1—O2	1.4438 (11)	C2—C1	1.507 (2)
S1—O3	1.4754 (11)	C2—H2A	0.9700
S1—O1	1.5553 (12)	C2—H2B	0.9700
S2—O8	1.4378 (14)	C5—C6	1.377 (2)
S2—O6	1.4387 (12)	C5—C10	1.380 (2)
S2—O7	1.4647 (12)	C1—H1C	0.9700
S2—O5	1.5542 (12)	C1—H1D	0.9700
N2—C5	1.4799 (18)	C4—H4A	0.9700
N2—C2	1.5027 (19)	C4—H4B	0.9700
N2—C3	1.5041 (18)	C10—C9	1.388 (2)
N2—H2	0.9100	C10—H10	0.9300
N1—C4	1.485 (2)	C6—C7	1.383 (2)
N1—C1	1.491 (2)	C6—H6	0.9300
N1—H1A	0.9000	C9—C8	1.373 (3)
N1—H1B	0.9000	C9—H9	0.9300
O1—H1	0.8200	C8—C7	1.383 (3)
O5—H5	0.8200	C8—H8	0.9300
C3—C4	1.507 (2)	C7—H7	0.9300
C3—H3A	0.9700		
			
O4—S1—O2	115.31 (8)	C1—C2—H2A	109.4
O4—S1—O3	111.75 (7)	N2—C2—H2B	109.4
O2—S1—O3	110.49 (7)	C1—C2—H2B	109.4
O4—S1—O1	108.60 (8)	H2A—C2—H2B	108.0
O2—S1—O1	104.35 (7)	C6—C5—C10	122.13 (15)
O3—S1—O1	105.60 (7)	C6—C5—N2	117.99 (13)
O8—S2—O6	115.46 (9)	C10—C5—N2	119.87 (13)
O8—S2—O7	111.29 (8)	N1—C1—C2	109.67 (12)
O6—S2—O7	110.95 (7)	N1—C1—H1C	109.7
O8—S2—O5	107.90 (8)	C2—C1—H1C	109.7
O6—S2—O5	103.68 (7)	N1—C1—H1D	109.7
O7—S2—O5	106.89 (8)	C2—C1—H1D	109.7
C5—N2—C2	111.93 (11)	H1C—C1—H1D	108.2
C5—N2—C3	112.96 (11)	N1—C4—C3	109.71 (12)
C2—N2—C3	109.52 (11)	N1—C4—H4A	109.7
C5—N2—H2	107.4	C3—C4—H4A	109.7
C2—N2—H2	107.4	N1—C4—H4B	109.7
C3—N2—H2	107.4	C3—C4—H4B	109.7
C4—N1—C1	111.13 (12)	H4A—C4—H4B	108.2
C4—N1—H1A	109.4	C5—C10—C9	118.29 (17)
C1—N1—H1A	109.4	C5—C10—H10	120.9
C4—N1—H1B	109.4	C9—C10—H10	120.9
C1—N1—H1B	109.4	C5—C6—C7	118.74 (17)
H1A—N1—H1B	108.0	C5—C6—H6	120.6
S1—O1—H1	109.5	C7—C6—H6	120.6
S2—O5—H5	109.5	C8—C9—C10	120.33 (18)
N2—C3—C4	109.89 (12)	C8—C9—H9	119.8
N2—C3—H3A	109.7	C10—C9—H9	119.8
C4—C3—H3A	109.7	C9—C8—C7	120.56 (18)
N2—C3—H3B	109.7	C9—C8—H8	119.7
C4—C3—H3B	109.7	C7—C8—H8	119.7
H3A—C3—H3B	108.2	C8—C7—C6	119.95 (18)
N2—C2—C1	110.98 (12)	C8—C7—H7	120.0
N2—C2—H2A	109.4	C6—C7—H7	120.0

### Hydrogen-bond geometry (Å, °)

**Table:**

*D*—H···*A*	*D*—H	H···*A*	*D*···*A*	*D*—H···*A*
O1—H1···O3^i^	0.82	1.80	2.6140 (17)	172
O5—H5···O7^ii^	0.82	1.80	2.6066 (18)	169
N1—H1A···O2^iii^	0.90	2.23	2.8636 (19)	128
N1—H1A···O3^iv^	0.90	2.30	3.0279 (19)	138
N1—H1B···O3	0.90	2.14	2.9251 (18)	145
N1—H1B···O2^i^	0.90	2.35	2.9892 (18)	128
N2—H2···O7	0.91	2.02	2.8216 (16)	146
N2—H2···O6^ii^	0.91	2.32	2.9037 (17)	122
C1—H1C···O8	0.97	2.59	3.500 (2)	156
C1—H1D···O2^i^	0.97	2.60	3.113 (2)	114
C3—H3A···O6^ii^	0.97	2.42	2.949 (2)	114
C3—H3B···O4	0.97	2.59	3.513 (2)	159
C6—H6···O7	0.93	2.55	3.246 (2)	132
C10—H10···O4	0.93	2.60	3.520 (2)	170

Symmetry codes: (i) *x*, −*y*+3/2, *z*−1/2; (ii) *x*, −*y*+1/2, *z*+1/2; (iii) −*x*+2, *y*−1/2, −*z*+3/2; (iv) −*x*+2, −*y*+1, −*z*+1.
